# Hydrogen Sulphide-Based Therapeutics for Neurological Conditions: Perspectives and Challenges

**DOI:** 10.1007/s11064-023-03887-y

**Published:** 2023-02-10

**Authors:** Amir H. Sharif, Mohammed Iqbal, Bahareh Manhoosh, Negin Gholampoor, Dan Ma, Mandeep Marwah, Lissette Sanchez-Aranguren

**Affiliations:** 1 College of Health and Life Sciences, Aston Medical School, Aston University, Birmingham, UK; 2 College of health and Life Sciences, School of Biosciences, Aston University, Birmingham, UK; 3 Translational Medicine Research Group, Aston Medical School, Aston University, Birmingham, UK

**Keywords:** Central nervous system, Hydrogen sulphide, Hydrogen sulphide-donating compounds

## Abstract

Central nervous system (CNS)-related conditions are currently the leading cause of disability worldwide, posing a significant burden to health systems, individuals and their families. Although the molecular mechanisms implicated in these disorders may be varied, neurological conditions have been increasingly associated with inflammation and/or impaired oxidative response leading to further neural cell damages. Therefore, therapeutic approaches targeting these defective molecular mechanisms have been vastly explored. Hydrogen sulphide (H_2_S) has emerged as a modulator of both inflammation and oxidative stress with a neuroprotective role, therefore, has gained interest in the treatment of neurological disorders. H_2_S, produced by endogenous sources, is maintained at low levels in the CNS. However, defects in the biosynthetic and catabolic routes for H_2_S metabolism have been identified in CNS-related disorders. Approaches to restore H_2_S availability using H_2_S-donating compounds have been recently explored in many models of neurological conditions. Nonetheless, we still need to elucidate the potential for these compounds not only to ameliorate defective biological routes, but also to better comprehend the implications on H_2_S delivery, dosage regimes and feasibility to successfully target CNS tissues. Here, we highlight the molecular mechanisms of H_2_S-dependent restoration of neurological functions in different models of CNS disease whilst summarising current administration approaches for these H_2_S-based compounds. We also address existing barriers in H_2_S donor delivery by showcasing current advances in mediating these constrains through novel biomaterial-based carriers for H_2_S donors.

## Introduction

Neurological disorders may result from insult to the brain, spinal cord or peripheral nerves as well as congenital defects, degeneration or structural defects. These impairments are increasingly recognised as major burdens to the affected patient, families and health systems as they are listed as a major cause of disability and second leading cause of death [1]. Neurological disorders, especially those with a degenerative nature, including Alzheimer’s disease (AD), Parkinson’s disease (PD) and Huntington’s disease (HD), are incurable, debilitating conditions resulting in progressive damage of nerve cells. In 2006, the Global Burden of Disease (WHO) estimated that combined, the prevalence of PD, AD and other dementias was estimated to reach 5.25 (per 1000 individuals) in 2015, however, these figures are projected to increase to 6.47 (per 1000) by 2030 [2] with all neurological-related cases predicted to triple in the coming decades to 152 million worldwide [1]. Therefore, improving therapeutic management options is of critical importance.

Hydrogen sulphide (H_2_S) is an endogenous gas which, along with nitric oxide and carbon monoxide, belongs to a family of transmitters known as gaseous transmitters [3]. H_2_S has increasingly gained recognition as a protective agent. Studies have shown that H_2_S is involved in a variety of physiological processes in the body, able to exert cytoprotective effects owing to its antioxidant properties and ability to modulate oxygen consumption [4]. However, H_2_S was once considered a toxic molecule. It has been demonstrated that H_2_S has a biphasic effect; low concentrations are beneficial, allowing enhanced mitochondrial respiration whereas higher concentrations inhibit the respiratory chain at level of mitochondrial complex IV, resulting in cytotoxicity [5].

In neurodegenerative conditions, the use of H_2_S-releasing compounds have demonstrated neuroprotective roles [6–8] when administered at physiological levels (approximately 20 to 300 µM) [9]. Although many molecular mechanisms have been described, our understanding of the potential for H_2_S-based compounds in the treatment or prevention of neurological disorders is not yet clear. Many of the challenges yet to be addressed include the rapid release of H_2_S and fast metabolism in vivo, the ability of H_2_S-related compounds to cross the blood brain barrier (BBB) and the optimisation of laboratory techniques to measure H_2_S levels in tissues or cells.

This literature review aims to highlight current evidence demonstrating the role of H_2_S in the central nervous system (CNS) whilst exploring the neuroprotective potential of H_2_S-donating compounds in different models of neurological disease, from in vitro to in vivo settings. Additionally, this review will explore challenges in H_2_S delivery to the nervous system and showcase potential approaches to mitigate these challenges.

## H_2_S in the Central Nervous System

### Synthesis of H_2_S

H_2_S is produced enzymatically by the action of three enzymes: cystathionine-β-synthase (CBS), cystathionine-γ-lyase (CSE), and 3-mercaptopyruvate (3-MST) [10]. CBS catalyses a β-replacement reaction between homocysteine and cysteine, producing cystathionine and H_2_S [11]. While CSE employs a similar method, it can also catalyse a reaction that converts cystathionine into cysteine, which can then be used in further reactions to produce H_2_S [12]. Furthermore, 3-MST is a mitochondrial enzyme that produces H_2_S by transferring sulphur from 3-meracaptopyruvate (produced by cysteine aminotransferase) to sulphurous acid. This produces thiosulfate as one of the products, which is reduced into H_2_S [13].

**Fig. 1:**
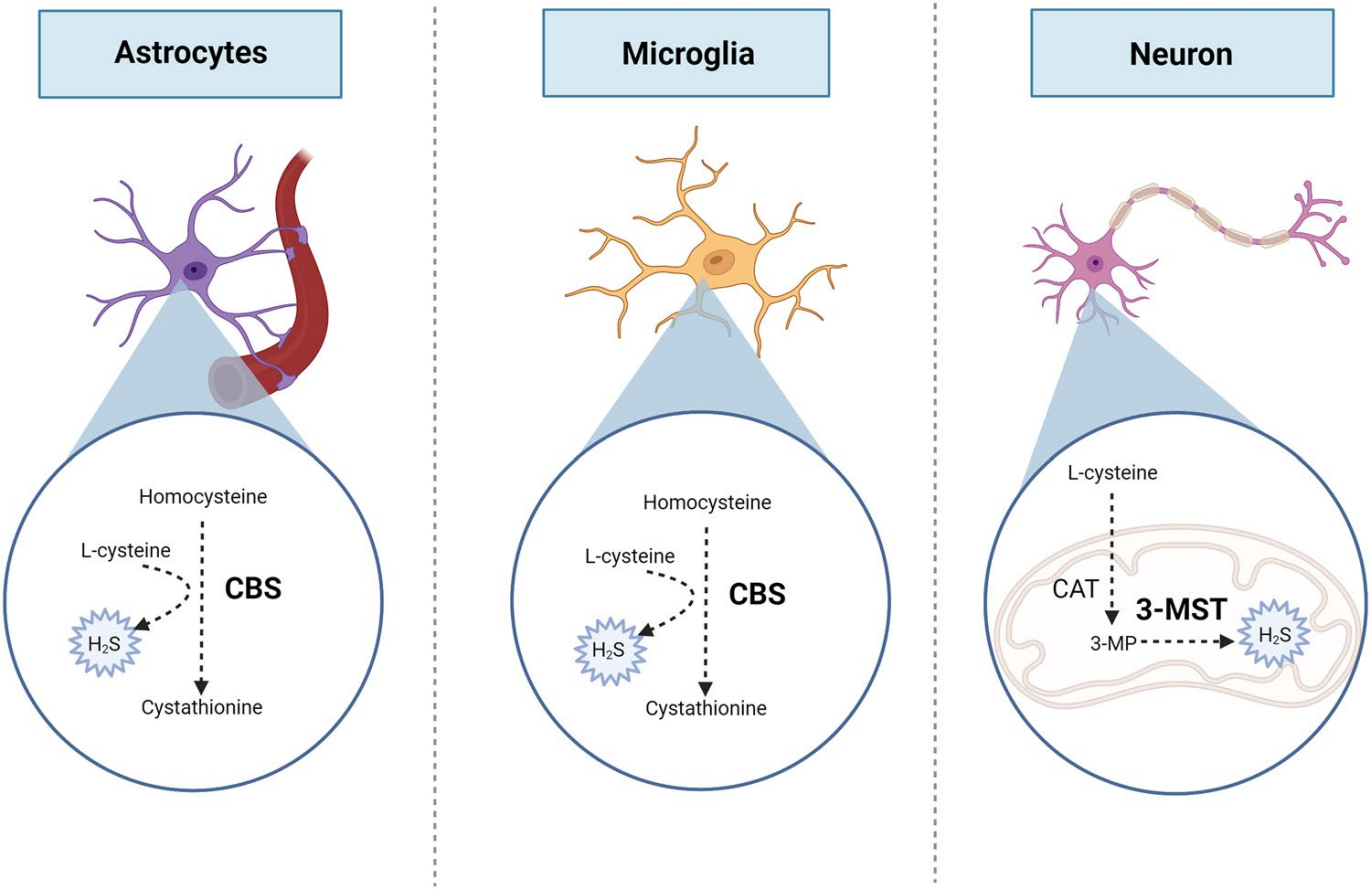
Synthesis of H_2_S in CNS-related cells. Cytoplasmatic cystathionine-β-synthase (CBS) expressed in astrocytes and microglia, and mitochondrial 3-mercaptopyruvate (3-MST) expressed in neurons, are mainly involved in the endogenous generation of H_2_S. CBS catalyse the reaction between homocysteine and cysteine, producing cystathionine and H_2_S whereas 3-MST produces H_2_S by transferring sulphur from 3-meracaptopyruvate (3-MP) (produced by cysteine aminotransferase, CAT) to sulphurous acid

In the CNS, CBS and 3-MST are the main enzymes involved in H_2_S production, with 3-MST being responsible for approximately 90% of the H_2_S produced in the brain [14]. While 3-MST is present mainly in neurons, CBS is present within astrocytes and microglia, suggesting that each enzyme may have a distinct role to promote H_2_S signalling [15]. Given that 3-MST is a located within the mitochondrial matrix [16], it highlights the potential role of mitochondrial H_2_S in regulating degenerative CNS processes whilst CBS has been found accountable for defective neurological functions such as neurogenesis, synaptogenesis and controlling BBB permeability [17]. Interestingly, CSE is hardly detected in brain tissue. Very low levels of CSE mRNA have been detected in the brain whilst CSE inhibitors have shown no significant suppression of H_2_S production [18], suggesting that CSE does not play a key role as a H_2_S producing enzyme in the brain (Fig. 1).

The importance of both CBS in generating H_2_S in brain cells (mainly astrocytes and microglia) suggest that their patterns of expression and/or activity may have an impact in the development of brain-related disorders. In neurodegenerative disorders such as PD and AD, hyperhomocysteinemia has been regarded as risk factor for the development of these conditions. Although accumulation of amino acid homocysteine leading to hyperhomocysteinemia may occur due to defects in different enzymes, evidence shows that CBS 844ins68 mutation and VNTR polymorphisms of the CBS gene are independent risk factors for AD development in subjects aged 75 years or more [19]. Moreover, recent evidence by Bjørke-Monsen, et al. 2022 showed that severe hyperhomocysteinemia in a patient with PD associated with reduced availability of CBS cofactor, pyridoxal 5-phosphate (PLP) [20]. These observations suggest that deficient CBS signalling, due to deficient availability of cofactor PLP and polymorphisms of CBS may be considered risk factors for the development of neurodegenerative diseases.

There are also non-enzymatic routes for H_2_S synthesis. These routes are usually coupled to reducing reactions such as glycolysis, which produces glucose and reducing equivalents such as nicotinamide adenine dinucleotide. These reducing molecules can then participate in reducing numerous sulphur compounds into H_2_S [21, 22].

### Catabolism of H_2_S

H_2_S is mainly metabolised through oxidation reactions in the mitochondria known as the “sulphur oxidation unit” pathway [23]. A major enzyme in this pathway is sulphide quinone oxidoreductase (SQR), which oxidizes H_2_S to persulfide. SQR is found in many organs such as the liver and colon. SQR protein and mRNA are expressed in mouse primary microglia and astrocytes but not in primary neurons [24]. The patterns of SQR expression shows lower levels of SQR mRNA and protein in the brain when compared to other tissues, suggesting that SQR may have limited contribution to the breakdown of H_2_S in the brain [22, 25].

Nevertheless, due to the remarkable importance of SQR in the metabolism of H_2_S in other tissues, researchers have investigated the presence of homolog forms of SQR in neuronal tissue. Ackermann et al. demonstrated the presence of a SQR homolog known as SQRDL (sulphur quinone oxidoreductase-like protein), which metabolises sulphur in rat brain [26]. This study also revealed that the SQRDL mRNA increased in rats with age, which may result in excess H_2_S degradation, contributing to age-related neurodegenerative disorders [26]. More research is required to better understand the existence of other relevant SQR variants that may play a significant role in the degradation of H_2_S.

**Fig. 2:**
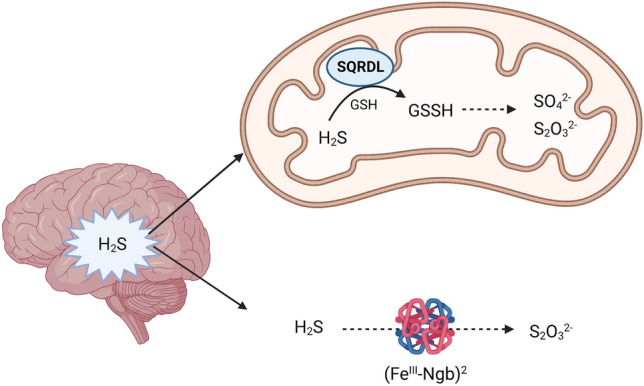
The oxidation (catabolism) of H_2_S in the brain is primarily mediated by the mitochondrial sulphide quinone oxidoreductase homolog, SQRDL (sulphur quinone oxidoreductase-like protein) generating sulphate (SO_4_^2−^) and thiosulphate (S_2_O_3_^2−^). Other routes of catabolism include the generation of thiosulphate (S_2_O_3_^2−^) from the reaction of H_2_S with ferrous neuroglobin (Fe^III^–Ngb)^2^

Other routes of H_2_S metabolism suggest the neuroglobin may have a role in H_2_S oxidation in the brain. Neuroglobin is a member of the haemoprotein family which uses its Fe^2+^ ion to bind to oxygen [27]. Neuroglobin binds to H_2_S and oxidises it to thiosulfate, however it has been suggested that this reaction is less efficient, compared to its haemoglobin and myoglobin counterparts [28]. In addition, as neuroglobin expression decreases with age in rodents, it has been suggested that it may play a role in age-related neurodegeneration [29] (Fig. 2). These association is still not yet well explored.

Altogether, the interplay between SQR and its homolog SQRDL, along with neuroglobin in the metabolism of H_2_S in the brain is not yet well established. Because SQR mRNA and protein expression is low in the brain, pathways involving neuroglobin and/or alternative SQR variants, warrant further investigation to better understand their role in sustaining H_2_S oxidation in the brain.

## Neuroprotective Mechanisms of H_2_S

New avenues of research have resulted in the development and refinement of several H_2_S donor compounds. These donors have been useful to elucidate key molecular mechanisms mediated by H_2_S in the context of physiological responses. Moreover, they have also allowed the exploration of their potential to modulate impaired responses and act as potential therapeutics in a myriad of human conditions. Table 1 summarises identified molecular mechanisms of H_2_S in diverse models of neurological conditions.

### H_2_S as ROS Scavenger

H_2_S is involved in a variety of physiological processes in the nervous system [10]. Kimura et al. demonstrated the protective effect of H_2_S in the nervous system, showing H_2_S is able to protect neuronal cells against oxidative stress (imbalance between oxidative molecules production and antioxidant mechanisms) by enhancing the synthesis of the antioxidant substrate glutathione (GSH) which functions as a storage for cysteine [8]. Importantly, GSH is maintained at high levels in astrocytes [8], where also CBS and SQR have been identified. In environments mimicking oxidative stress, such as in the presence of hydrogen peroxide (H_2_O_2_), H_2_S has also demonstrated the ability to reduce oxidative damage in mouse brain neuroblastoma cells Neuro2a, by enhancing GSH levels reduced by H_2_O_2_ [30]. It is possible that the regulation of sulphur/GSH metabolism in astrocytes is a key regulatory mechanism for the protection against oxidative molecules in the CNS. In addition to the enhanced production of GSH resulting from H_2_S, other mechanisms involving the direct scavenging of reactive oxygen species (ROS) by H_2_S have been reported. In this regard, H_2_S donor NaHS, has been identified as a direct scavenger of peroxynitrate, a reactive molecule derived from nitrogen in neuroblastoma cells SHSY-5Y [31].

### Anti-apoptotic Role of H_2_S

Apoptosis, or programmed cell death, may occur through the intrinsic (mediated by the mitochondria) or extrinsic (associated with death receptor) pathways [32]. H_2_S has been shown to exert anti-apoptotic effects through the intrinsic pathway linked to its ROS neutralising properties, thus preventing oxidative stress to neuronal cells [33]. H_2_S can exert its anti-apoptotic effect via modulating nuclear translocation of nuclear factor kappa B (NF- κB) which is a transcription factor that can activate anti-apoptotic genes [34]. Additionally, the administration of NaHS has been observed to abrogate the generation of H_2_O_2_, reducing hippocampal neuronal apoptosis in mouse [35]. Studies by Shan et al., showed pre-treatment with H_2_S donors in a model of intracerebral haemorrhage in mice, significantly reduced caspase 3 and Bcl-2 (regulators of apoptosis) [36]. The molecular mechanisms linking anti-apoptotic function and ROS neutralising effects might work in parallel in the presence of H_2_S and this evidence suggest that H_2_S therapies may be effective to ameliorate a broad spectrum of pathophysiological mechanisms underpinning neurological conditions.

### Role of H_2_S in Neuroinflammation

H_2_S has been shown to play a vital role in preventing neuroinflammation. Damage to neuronal cells can result in neuroinflammation by activation of pro-inflammatory cytokines that can aggravate the inflammation leading to neuronal death [37]. Several reports have demonstrated that H_2_S is able to prevent neuroinflammation by modulating the inflammatory response. In this regard, NaHS, has been observed to modulate inflammation by inhibiting the release of tumour necrosis factor- α (TNF-α) and reducing the expression of toll-like receptor 4 (TLR4), and NF-κB in the hippocampus of Sprague-Dawley rats subject to subarachnoid haemorrhage [38]. Furthermore, in rat models of AD established by injections of Amyloid-β1–40 into the hippocampus, H_2_S significantly reduced the release of inflammatory cytokines interleukin IL-1β and TNF-α whilst ameliorating spatial learning and memory impairment [39]. These observations suggest an interesting link between neuroinflammation and cognitive impairments that might be ameliorated by H_2_S.

**Table 1:** Molecular mechanisms of H_2_S action explored using in vitro and in vivo models of neurological diseases

Disorder	H_2_S protective effects	Results/evidence
Parkinson’s disease	Antioxidant Cytoprotectant	• Inhibition of ROS-RNS levels [40]^a^ • Elevation of GSH and superoxide dismutase (SOD) [40, 41]^a^ • Enhanced expression of antioxidant enzymes [41]^a^ • Suppression of MDA [41]^a^ • Reduced cytotoxicity [40, 42]^a,b^ • Decreased caspase-3 [40] ^a^ and 12-induced apoptosis [43]^b^ • Decreased Bcl-2/Bax ratio [40]^a^ • Restoration of mitochondrial membrane potential [40]^a^ • Neurogenesis via the Akt/GSK-3β/β-catenin pathway [42]^b^ • Sulfhydration of Parkin [44]^a^
Alzheimer’s disease	Antioxidant Cytoprotectant Anti-inflammatory	• Inhibition of ROS and RNS levels [45]^b,c^ • Sulfhydration of GSK3β [46]^c^ • Reduced hyperphosphorylation of tau protein [46, 47]^c^ • Decreased size of β-amyloid plaques [47, 48]^c,a^ • Mitochondrial DNA integrity [49]^c^ • Decreased caspase 3 activation [45]^b,c^ • Decreased Bcl-2/Bax ratio [45]^b,c^ • Reduction of TNF-α levels [45, 50]^b,c^
Huntington’s disease	Antioxidant Cytoprotectant Anti-inflammatory	• Reduced MDA levels [51]^b^ • Enhanced expression of the Nrf2 antioxidant gene [51]^b^ • Decreased caspase 3 activation and cytochrome c levels [51] ^b^ • Reduction of TNF-α levels [51]^b^
Stroke	Cytoprotectant Anti-inflammatory	• Reduction of ischemic/reperfusion injury [52–54]^b^ • Reduced risk of haemorrhagic stroke [55]^b^ • Suppression of pro-inflammatory cytokine levels [54, 56]^b^
Brain tumours	Cytotoxic Pro-apoptotic Anti-apoptotic	• Induction of DNA damage [57]^a^ • Sensitization to proton and photon radiation [57]^a^ • Induction of caspase-dependent apoptotic pathway [58, 59]^a^ • Increased Bcl-2/Bax ratio [58, 59]^a^ • Decreased Bcl-2/Bax ratio [60]^a^ • Tumour growth [61]^b^
Epilepsy	Cytoprotectant Cytotoxic Anti-inflammatory	• Reduction of seizures [62]^b^ • Aggravation of seizure events [63]^b^ • Decreased levels of pro-inflammatory markers and cytokines [62, 64]^b^ • Increased anti-inflammatory cytokines (IL-10) [62]^b^
Multiple sclerosis	Anti-inflammatory	• Upregulation of anti-inflammatory cytokines [65]^a^ • Inhibition of adhesion molecules [65]^a^ • Reduced migration of peripheral blood mononuclear cells [65]^a^ • Upregulation of TGF-β in dendritic cells [66]^a^ • Reduced IL-17 and IFN-γ [66]^a^

Models of disease: ^a^In vitro

^b^In vivo, chemically induced

^c^In vivo, genetically-induced

## CNS-Related Disorders and H_2_S

### Parkinson’s Disease (PD)

PD is one of the most prevalent neurodegenerative disorders worldwide. Motor symptoms of Parkinson’s include bradykinesia, resting tremor, and cogwheel rigidity whereas non-motor symptoms include sleep disturbance, cognitive decline, and depression [67]. The pathophysiology of PD involves the progressive destruction of the dopaminergic neurons in the nigrostriatal pathway in the midbrain [68]. Accumulation of ROS can damage the DNA leading to oxidative stress and cell death are associated with PD [69].

Several models of PD have been proposed in order to better understand this disorder. Examples include the use of the neurotoxin 1-methyl-4-phenyl-1,2,3,6-tetrahydropyridine (MPTP) and its active metabolite 1-methyl-4-phenylpyridinium (MPP+). MPP^+^ plays an active role in destroying the dopaminergic neurons that results in parkinsonism [70]. An in vitro study used the human neuroblastoma cells (SH-SY5Y) to investigate the role of H_2_S in attenuating the oxidative stress on the neurons in PD cell model [40]. This study used an MPP^+^-treated SH-SY5Y cell model showing that NaHS increased the cell viability, reduced cytotoxicity, and reduced oxidative stress-induced cell apoptosis in a dose dependent manner in the MPP^+^ treated cells [40].

Another study investigated the neuroprotective role of H_2_S in a mouse model of PD. In line with the study carried out by Liu et al. [40], this study also showed that H_2_S elicited neuroprotective properties. MPTP was used in this study to generate a mouse model of PD and NaHS was used to investigate the effect of H_2_S. This study demonstrated that H_2_S prevents MPTP-induced neuronal damage and promotes neurogenesis through Akt/glycogen synthase kinase-3β (GSK-3β)/β-catenin pathways in adult neuronal stem cells [42]. Consistently, in a similar model of MPTP-induced PD in mice established by Lu et al., it was observed that NaHS reduced primary mesencephalic neurons cytotoxicity whilst explored molecular mechanism associated highlighting that H_2_S was able to enhance mitochondrial uncoupling protein 2 (UCP2) antioxidation resulting in abrogated ROS generation and reduced caspase 12-induced apoptosis [43]. Mitochondria are the main site of ROS generation thus may play a key role in the development of PD whilst compounds modulating mitochondrial-ROS, such as H_2_S, may be potential therapeutic candidates in the treatment of PD.

**Fig. 3:**
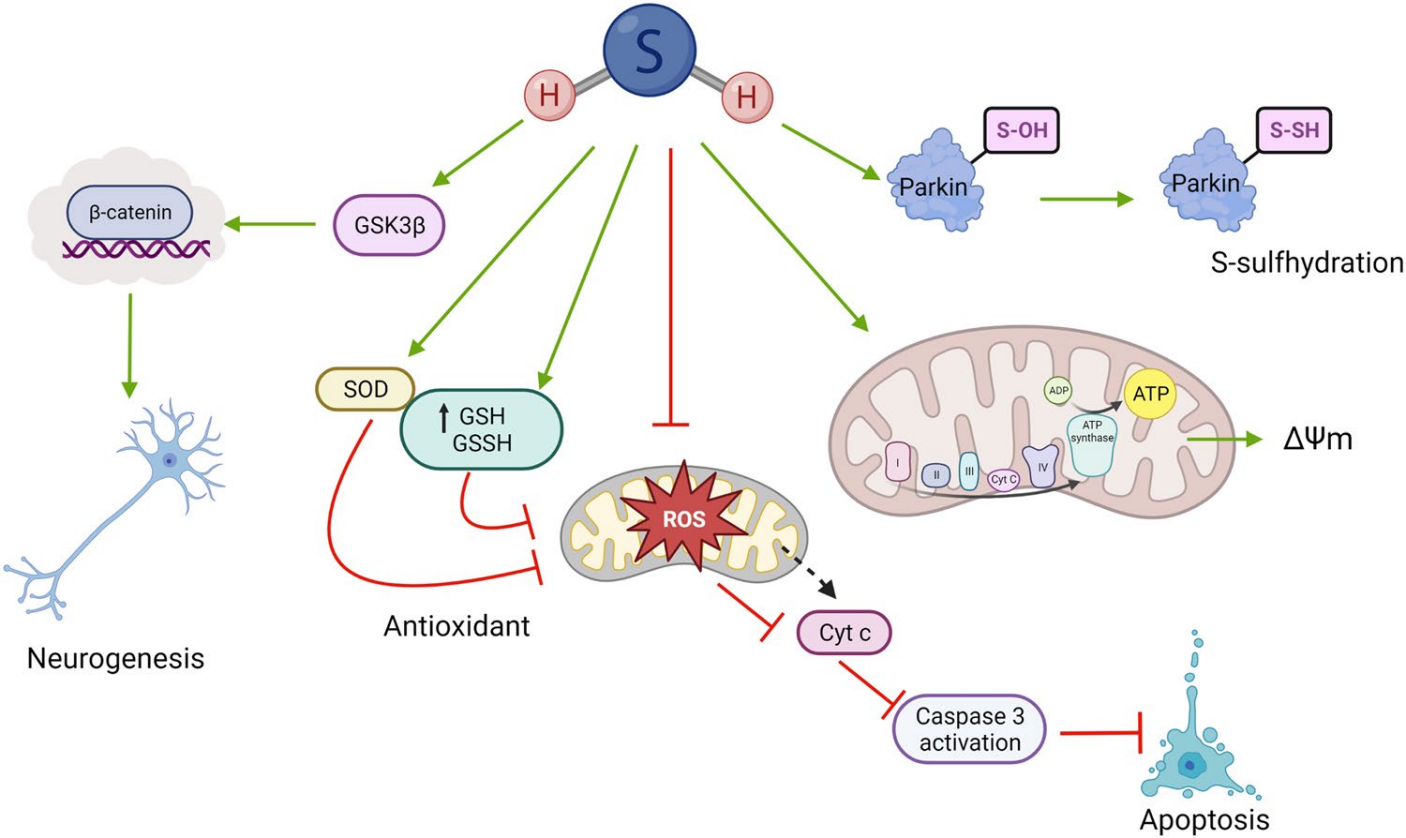
Protective molecular mechanisms of H_2_S in Parkinson’s disease (PD). The neuroprotective effects of H_2_S in PD are associated with neurogenesis mediated by GSK3β/β-catenin pathway. H_2_S has antioxidant properties observed by the induction of antioxidant defence mediated by upregulation of superoxide dismutase (SOD) and glutathione (GSH). H_2_S can also reduce levels of reactive oxygen species (ROS) acting as a direct scavenger or indirectly by restoring the mitochondrial membrane potential (ΔΨm). The reduction in accumulation of mitochondrial-ROS results in inhibition of apoptosis mechanisms via caspase 3. Moreover, H_2_S may also regulate parkin functions by mediating posttranslational modifications of active cysteine residues, a process known as S-sulfhydration or persulfidation

Although the molecular mechanisms of H_2_S-induced neuroprotection in PD may be broad (Fig. 3), these new avenues in PD research position H_2_S as a potential mitochondrial protectant. Thus, H_2_S may be a suitable therapeutic candidate for clinical use in treating Parkinson’s in the future.

### Alzheimer’s Disease (AD)

AD is the most common neurodegenerative disorder and is the leading cause of dementia in the elderly population. Oxidative stress, neuroinflammation and damage to cholinergic neurons are some of the pathophysiological changes that are involved in AD [71].Furthermore, the accumulation of the microtubule-associated protein Tau [72] and β-amyloid peptides in neuronal cells [73] have been proposed as key molecular mechanism leading to this devastating disorder.

A research study investigated the neuroprotective role of H_2_S in an animal model of AD by using the 3xTg-AD mouse model which includes Tau protein mutations: PS1M146V, APPSwe, and Tau P301L [46]. This study showed that H_2_S prevents hyperphosphorylation of the Tau protein by S-sulfhydration (post-translational modification of proteins mediated by H_2_S) of GSK3β. Additionally, it was observed that the CSE catalytic effect was augmented by binding wild type Tau which resulted in decreased levels of Tau protein in the cell. In contrast, CSE could not bind the mutated Tau P301L. Consequently, CSE levels were decreased in the cortex and hippocampus of the brain in the 3xTg-AD mouse model compared to wild type. This interesting report demonstrated a direct relationship between decreased endogenous levels of H_2_S and AD-like condition in vivo. Interestingly, it was demonstrated that administrating H_2_S donor, GYY4137, by daily intraperitoneal injection for 12 weeks (100 mg/kg) to 3xTg-AD mice improved their motor and cognitive function. The level of Tau protein S-sulfhydration was higher in the group treated with GYY4137 compared to the control group [46].

Aligned with the study carried out by Giovinazzo et al. [46], a study by Vandini et al. [47] using 3xTg-AD transgenic mouse model, previously showed that intraperitoneal injection of sulphur water and NaHS daily for three consecutive months improved memory and cognitive functions in both young and aged animals Additionally, treatment with NaHS and sulphur water in 3xTg-AD mouse decreased the size of the β-amyloid plaques in the cortex and hippocampus. The molecular mechanisms implicated suggest the inhibition of c-Jun N-terminal kinases, extracellular signal-regulated kinases, and p38 protect against neuroinflammation and Tau protein hyperphosphorylation, leading to a decrease in accumulation of β-amyloid plaques in the cortical and hippocampal regions of the brain [47].

Moreover, a recently synthesised H_2_S donor targeted to the mitochondria, AP39, was probed against a model of AD in mice (APP/PS1 double-transgenic mice) [49]. AP39 injected intraperitoneally for 6 months enhanced cellular bioenergetic function and had mitochondrial protective effect on AD neurons and mice. Neurons treated with AP39 showed reduced ROS levels and increased ATP production. Within the AD neurons, it was observed that mitochondrial DNA was damaged, however AP39 treatment prevented these alterations by increasing mitochondrial DNA integrity. Furthermore, APP/PS1 double-transgenic mice treated with AP39 observed an improvement in their learning and memory impairments [49] suggesting that H_2_S, targeted to the mitochondria, could be a potential therapeutic candidate for AD. Given the fact that accumulation of Tau protein disturbs the neuronal mitochondrial respiration, molecular mechanism implied may include the restoration of the flow of electrons in the oxidative phosphorylation and/or antioxidant effects abrogating neurotoxicity induced by Tau, however, these theories warrant further exploration.

**Fig. 4:**
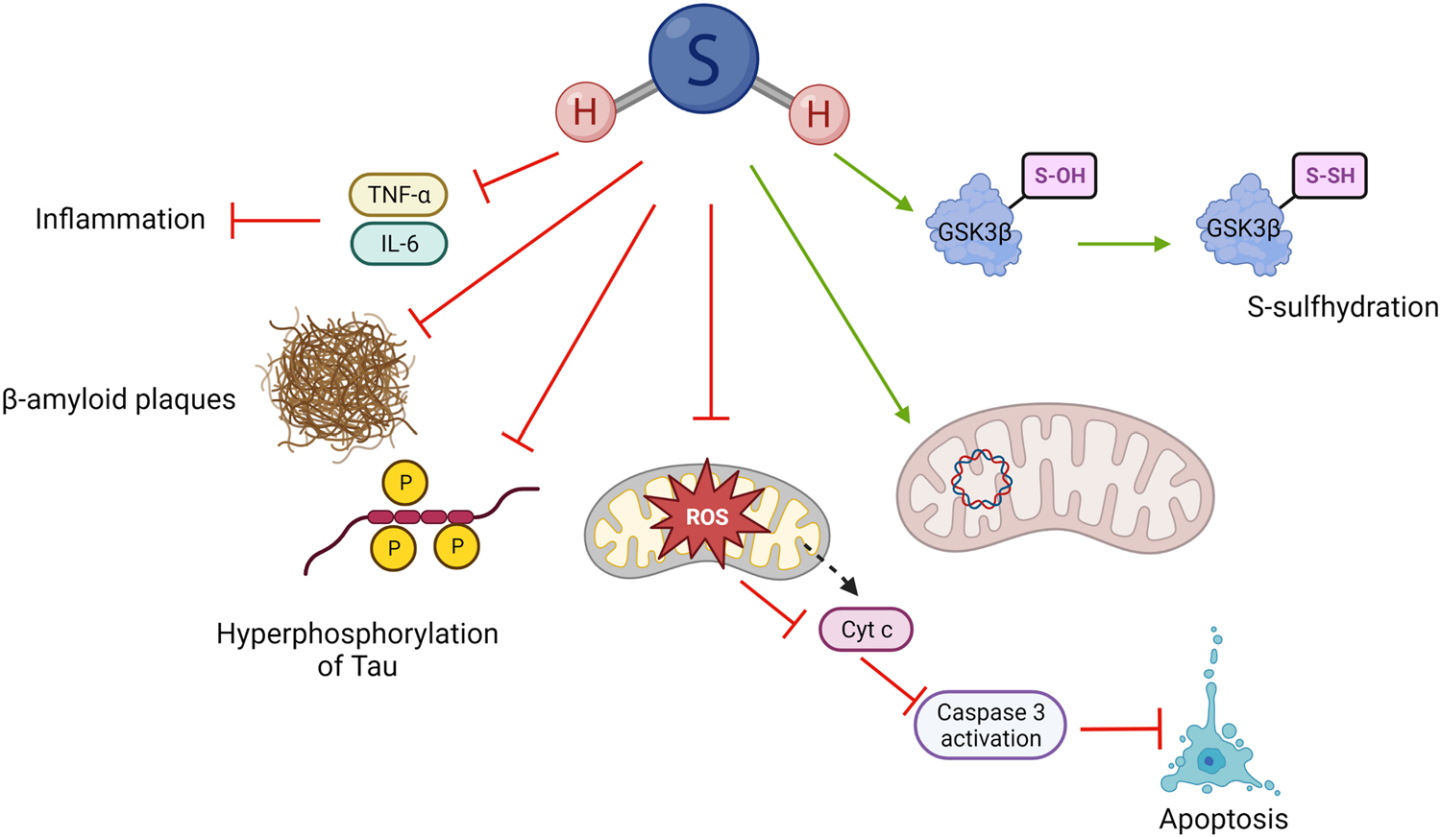
Protective mechanisms of H_2_S in AD. The neuroprotective effects of H_2_S in AD are associated with inhibition of anti-inflammatory cascade via reduction of TNF-β and IL-6 levels. Moreover, H_2_S has been linked to reduction of size of β-amyloid plaques and reduction in hyperphosphorylation of Tau. H_2_S has been observed to reduce levels of mitochondrial reactive oxygen species (ROS) leading to inhibition of apoptosis via caspase 3. H_2_S may also maintain the integrity of mitochondrial DNA whilst it may also regulate GSK3β protein functions via S-sulfhydration (persulfidation)

Overall, H_2_S has proven to play a significant neuroprotective role in AD models (Fig. 4). However, the translation of these results to the clinical setting might be hindered by complex dosage regimen of the H_2_S donor, requiring multiple and extensive dose regimes (3 to 6 months) as observed in these studies [46, 47, 49]. This emphasises the necessity to further explore patient-friendly approaches including better delivery systems and/or more stable H_2_S donors to reduce frequency of administration.

### Huntington’s Disease (HD)

HD is an autosomal dominant progressive neurodegenerative disorder resulting from the toxic accumulation of mutant huntingtin protein in neurons [74]. The pathophysiology of HD involves the selective destruction of the corpus striatum in the brain which controls motor activities leading to involuntary and irregular muscle movements, behavioural changes and cognitive decline [75].

Paul et al. [76] explored the neuroprotective role of H_2_S in a mouse model of HD showing a direct correlation between reduction in CSE levels and increased neurodegeneration in the striatum. This study used both in vivo and in vitro models to demonstrate the impact of CSE depletion in the pathophysiology of HD. CSE depleted (CSE^−/−^) mice model exhibited abnormal hindlimb clasping and clenching resembling HD. Additionally, this study demonstrated that CSE level was significantly decreased in the striatal cell line of HD cell model consisting of 111 glutamine repeats (STHdhQ^*111/Q111*^(Q111) compared to the control group [76]. These observations suggest the importance of H_2_S in HD and imply the potential application of H_2_S donors in halting the progression of the disorder.

In an attempt to explore the potential for NaHS in abrogating HD-like effects induced by 3-nitropropionic acid (3NP), a study in a rat HD model revealed that H_2_S improved overall cognitive and locomotor deficits whist provided antioxidant, anti-inflammatory and anti-apoptotic effects observed by reduced levels of oxidative stress marker malondialdehyde (MDA), TNF-α and caspase 3 activation [51]. Interestingly, this study also revealed that treatment with NaHS significantly enhanced CBS expression, suggesting that H_2_S availability may have a crucial effect in modulating endogenous H_2_S pathways. Nonetheless, these assumptions require further exploration to understand the potential molecular mechanisms implicated.

### Cerebral Vascular Disease

Stroke is the second most prevalent cause of mortality worldwide. The two types of strokes include haemorrhagic and ischemic stroke [77]. Both lead to ischemia and consequent tissue damage/death.

Given the association between ischemic stroke and ROS, H_2_S as an antioxidant compound, may be a suitable candidate for management of these conditions. A study investigated the neuroprotective role of H_2_S in acute ischemic stroke using a model of cerebral ischemia/reperfusion injury in mice by administrating NaHS before reperfusion following an ischemic insult in the brain. By means of H-magnetic resonance spectroscopy/magnetic resonance spectroscopy (H-MRI/MRS) and immunohistochemistry, it was shown that the total infarct volume was reduced compared to sham, and administration of NaHS 1 min before perfusion exerted stronger effect that of 30 min [52]. Another study exploring the effect of NaHS on transient cerebral ischemia immediately after reperfusion, this, combined with mild hypothermia resulted in a decrease in ischemic-reperfusion injury via upregulation of N-methyl-D-aspartate receptor (NMDAR) [53].

To study mechanisms of haemorrhagic stroke in vivo, models such as the middle cerebral artery occlusion (MCAO) plus intravenous injection of tissue plasminogen activators (tPA), have been used [78]. Using this model, it was demonstrated that H_2_S reduces tPA-induced cerebral haemorrhage following MCAO. In this study, co-administration of two structurally distinct H_2_S donors, ADT-OH and NaHS reduced the tPA-induced cerebral haemorrhage through the inhibition of AKT-VEGF-MMP9 signalling cascade [55]. As one of the risk factors associated with tPA in the management of ischemic stroke, is the increased risk of cerebral haemorrhage, observations by Liu et al. [55] suggest that H_2_S donors may reduce the risk of haemorrhagic stroke in tPA-exposed subjects. Although more research is necessary to confirm this hypothesis, the co-administration of H_2_S and tPA may bring new avenues and may provide patients requiring treatment for ischemic stroke a better recovery profile.

### Brain Tumours

Brain tumours carry a high morbidity and mortality rate which highlights their clinical significance and burden to health systems worldwide. The most malignant brain tumour is glioblastoma multiform (GBM) which can rapidly spread and invade brain parenchyma [79]. Despite the growing number of treatment options available for the patients, brain malignancies carry a poor prognosis [80].

An in vitro study investigated the anti-cancer properties of H_2_S in C6 glioma cells [58]. It was shown that NaHS induces C6 glioma cell apoptosis via upregulation of caspase 3 and Bax proteins and downregulation of Bcl-2 protein through p38 MAPK signalling pathway [58]. In contrast to this report, another in vitro study demonstrated that H_2_S promotes C6 glioma cell growth. However, when CBS activity was compromised, the enhanced cell proliferation was blunted [60]. This study showed that cell apoptosis was reduced resulting in increased proliferation and viability rates of C6 glioma cells when exposed to NaHS suggesting that p38 MAPK/ERK1/2-COX-2 pathways are involved in NaHS-induced cancer cell proliferation and anti-apoptosis in C6 glioma cells [60]. An in vivo study reported by Li et al. showed that the size of the tumour was significantly increased in the rats exposed to NaHS in GBM group compared to the GBM only group, and H_2_S promotes C6 glioma cell growth via augmenting neurovascular formation and increasing hypoxia-inducible factor-1alpha (HIF-1α) expression [61]. These contrasting results more investigation is required to better understand the role of H_2_S in promoting/mitigating the expansion of brain tumours.

### Epilepsy

Epilepsy is one of the most common neurological disorders affecting around 50 million of the global population. This condition is characterised by recurrent seizures which is the clinical manifestation of alerted neuronal electrical activities [81]. Additionally, despite antiepileptic drugs offering many patients symptomatic relief, effective management is not achieved in almost one third of patients with current pharmacotherapies. Hence, new medical therapies for epilepsy are needed [82].

Pro- inflammatory cytokines such as IL-1β, IL-6, and TNF-α play an important role in the pathophysiology of the epileptic seizures and the level of these cytokines is raised in the serum and cerebrospinal fluid of epileptic patients [83]. Increased level of pro-inflammatory cytokines results in increased excitability of neurons which may be crucial in the pathophysiology epilepsy [84]. As H_2_S promotes the release of anti-inflammatory cytokines and is involved in preventing neuroinflammation [85], it might have a potential clinical application in the treatment of epilepsy.

The role of H_2_S in pilocarpine-induced status epilepticus (SE) in vivo has been explored. Adult male C57BL/6 mice were exposed to pilocarpine to induce SE whilst treatment consisted of exposure to a novel carbazole-based H_2_S donor. This study demonstrated that SE + H_2_S group had a shorter duration of seizure compared to the SE group. Additionally, this group showed delayed onset of seizure, reduced damage to the hippocampus and reduced pro-inflammatory state in microglia compared to the SE control group [62].

In contrast, a study performed by Luo et al. [63] showed that H_2_S delivered as NaHS exacerbates Pentylenetetrazole (PTZ)- and pilocarpine‐induced seizures in rats [63]. PTZ is a GABA-A receptor antagonist that when sequentially injected to animals, results in the development of chemical kindling, an epilepsy model [86]. Luo et al. [63] exposed rats to PTZ/pilocarpine and PTZ/pilocarpine + NaHS showing that the severity of seizures increased in the group treated with NaHS compared to the control group. Furthermore, the duration of seizure was longer in those rats exposed NaHS suggesting that H_2_S increased membrane excitability of entorhinal cortex neurones via facilitating the function of voltage‐gated sodium channel, AMPAR, and NMDAR [63]. These observations are not consistent about the effect of exogenous H_2_S on the manifestation of epilepsy. However, they suggest a potential role of H_2_S signalling in the modulation and severity of epilepsy. Further research is warranted to better understand the potential of the modulation of H_2_S signalling in the management of epilepsy.

### Multiple Sclerosis (MS)

MS is an autoimmune disease affecting the myelin structure in the CNS. Currently, 2.8 million people are affected worldwide, however, this number is on the rise [87]. MS results in a wide range of signs and symptoms that are variable depending on the severity and location of the lesions with optic neuritis being the most common [88]. Currently, the available immune-based therapies do not significantly improve MS prognosis, therefore, new treatment strategies, such as H_2_S donors, warrant exploration in the pursuit of better patient outcomes.

In physiological settings, the BBB protects the CNS by making it inaccessible to immune cells, while in MS, the BBB gets damaged due to endothelial cells’ impairment [89]. This results in an exacerbated influx of inflammatory mediated cells into the brain tissue, which induces demyelination and axonal dysfunction. Recurrent inflammatory responses in MS irreversibly damage the CNS nerve cells with the involvement of ROS [90]. The pathophysiology of MS also involves chronic platelet activation that promotes differentiation of autoreactive T cells resulting in initiation and progression of autoimmune neuroinflammation [91]. Therefore, two potential molecular mechanisms by which H_2_S may show potential beneficial effects in MS include its antioxidant role as well as its ability to inhibit platelet activation and adhesion molecule-mediated aggregation [92].

An in vitro study by Talaei et al. [65] examined the effect of NaHS on peripheral blood mononuclear cells (PBMNCs) obtained from healthy individuals to access its transmigration across an endothelial cells’ barrier representing the BBB. This study showed that pre-treatment of human endothelial cells with NaHS decreased PBMNC transmigration of both control and serotonin-treated cells. Additionally, treatment with NaHS resulted in upregulation of IL-10 and downregulation of adhesion molecules: lymphocyte function-associated antigen 1 (LFA-1) and vascular cell adhesion protein 1 (VCAM-1). This suggests that whilst NaHS resulted in relaxation/expansion of endothelial cells it may also cause morphological changes to the BBB leading to increased BBB permeability during MS attacks [65].

Lazarević et al. [66] studied the in vitro effect of H_2_S donor GYY4137 on differentiated dendritic cells and T cells which are both involved in the pathogenesis of MS. GYY4137 enhanced transforming growth factor β (TGF-β) expression in dendritic cells, suggesting an anti-inflammatory effect of GYY4137 in an MS-like environment. Additionally, isolated lymph node and spinal cord T cells were obtained from mice and rats immunised with CNS antigens and treated with GYY4137. GYY4137 significantly reduced interferon gamma (IFN-γ) and IL-17 production and reduced the proportion of FoxP3 + regulatory CD4 + T cells in the lymph node and spinal cord T cells. Interestingly, when the expression of H_2_S-producing enzymes (CBS, CSE, and 3-MST) was assessed in immune cells from healthy donors and drug-naïve relapse-remitting MS patients, the authors reported no differences in the CSE and CBS expression but lower expression of 3-MST [66].

Given the relevance of 3-MST in the generation and availability of H_2_S within the mitochondria, this study further suggests a potential link between H_2_S signalling and mitochondria in the modulation of anti-inflammatory response in MS. However, this theory awaits more exploration.

## Challenges in H_2_S Delivery to the Nervous System

Several limitations associated with the gaseous nature and rapid release of H_2_S from its various donor molecules hinder the translation of H_2_S-based therapeutics to the clinic. As shown throughout this review, it appears that a systemic increase in H_2_S availability may improve neurological function in certain neurological disorders such as PD and AD.

In conditions where H_2_S may have a therapeutic effect, desirable H_2_S-based compounds should bear long-circulating times with slow-releasing patterns. As Table 2 shows, current research evidence displaying the potential therapeutic effect of H_2_S donors in CNS related conditions, usually require extensive dosage regimes, ranging from weeks to months with daily administration of the donor, in order to observe therapeutic effect. This is consistent with previous observations that bolus administrations of H_2_S-donating compounds may result in spiked concentrations of H_2_S [93], leading to complex administration regimes. Understanding the burden that neurological conditions put on patients, families and healthcare professionals, patient-friendly approaches are necessary. This suggest that strategies to reduce administration of the compounds is of key importance. In an attempt to overcome this issue, several compounds with H_2_S-releasing properties have been developed which differ in their patterns of H_2_S release, including mechanisms triggered by hydrolysis, enzymatic reactions, pH variation, novel formulation strategies amongst others [94].

Another important limitation arises from the fact that to reach the brain, therapeutic compounds require to be able to cross the BBB. The BBB is a structure separating the blood and brain compartments. Its inner layer is composed of endothelial cells with tight junctions and its outer layer consists of pericytes and astrocytes [95]. The BBB allows hydrophobic substances (e.g., water and oxygen) to access the brain whilst preventing pathogens and large and hydrophilic substances (e.g., microorganisms and drugs) from entering the brain. For this reason, most drugs targeting the CNS cannot enter the brain, bringing difficulties in developing new efficient treatments for neurological diseases.

In terms of sulphide donating salts, ions diffuse passively across the BBB and their flow can be accelerated by partial association between anions and cations to form neutral ion-pair species in solution [96]. Thiosulfate does not permeate across cell membranes freely as it is an anion, therefore it must be transported by a transport mechanism to get into cells. The transport mechanism for thiosulfate is an SLC13 protein called sodium sulphate cotransporter 2 (SLC13A4, NaS-2), which is found within the brain. The transport mechanism helps the transportation of thiosulfate across the cell membrane [97], therefore, approaches targeting these transport mechanisms may offer a feasible method to enhance H_2_S delivery, when thiosulphate is employed. Therefore, along with developing targeted formulations, understating the chemistry of H_2_S-donating compounds is key in the successful delivery of these drugs to the CNS.

**Table 2:** Protective roles of H_2_S donors in neurological disease models

H_2_S donor	Relevant disease	Study approach	Regime of H_2_S donor treatment	References
ACS84	PD	1. SHSY-5Y exposed to 6-hydroxydopamine (6-OHDA) 2. Male Sprague-Dawley rats 6-OHDA hydrobromide was unilaterally injected into the left striatum.	1. ACS84 1 h prior the administration of 6-OHDA. 2. ACS84 (10 mg/kg/day) from the 5th to 7th week after 6-OHDA lesion.	[41]
ADT-OH	Stroke	Adult male C57BL/6J mice. 1. (MCAO) 2 h occlusion followed by reperfusion. 2. Hyperglycaemia MCAO model of tPA-enhanced haemorrhagic transformation.	ADT-OH (50 mg/kg) was administered at the onset of reperfusion or when the tPA infusion began	[55]
		Male ICR mice. MCAO was induced by an intraluminal monofilament technique.	ADT-OH (50 mg/kg/day) was administered after 3 h of reperfusion via intraperitoneal injection.	[54]
AP39	AD	1. Heterozygous APP/PS1 double-transgenic mice (APPswe-PS1dE9). 2. Primary cultures of cortical neurons from hemizygous transgenic mice and non-transgenic littermates.	1. AP39 (100 nM/kg) daily intraperitoneal injection for 6 weeks prior to the experiments. 2. Primary neurons were treated with AP39 (25–250 nM) for 24 h.	[49]
	Stroke	Male Sprague-Dawley rats subjected to MCAO.	Pre-treatment with AP39 50-nmol/kg during days, with the last dose 72 h before ischemia.	[98]
Carbazole-based H_2_S donor (C_15_H_14_N_2_S)	Epilepsy	Male Sprague–Dawley rats subjected to intraperitoneal injection of PTZ (40 mg/kg).	Carbazole-based H_2_S donor (500 µM) injected into the lateral ventricle, 1 h after PTZ administration.	[64]
Diallyl disulphide (DAD) and diallyl trisulfide (DAT)	GBM	Human T98G cells Human U87 cells	DAD (100 µM) for 24 h. DAT (25 µM) for 24 h.	[59]
		Ectopic glioblastoma Induction in NOD.CB17-prkdcscid/J mice by subcutaneous implantation of U87MG cells.	DAT (10ug/kg, 100ug/kg, and 10 mg/kg) 7 daily oral treatments for 4 weeks.	[99]
GYY4137	AD	3xTg-AD mice. These mice overexpress mutant human APP, PS1 and tau protein.	GYY4137 (100 mg/kg) injected intraperitoneally for 12 weeks.	[46]
	MS	1. C57BL/6 mice 2. Dark Agouti rats Immunised with 0.5 mg/mL guinea pig myeline oligodendrocyte glycoprotein. Lymph nodes were isolated after 7 days.	GYY4137 (200 µM) lymph nodes were cultured in the presence of GYY4137 from 40 min to 12 h.	[66]
	Stroke	Sprague–Dawley rats. Transient middle cerebral artery occlusion (tMCAO) model. 1.5 h ischemia and 24 h reperfusion	GYY4137 (1 mM in 5µL) was injected in the lateral cerebral ventricle just before reperfusion	[100]
Sodium hydrosulphide (NaHS)	PD	SHSY-5Y exposed to MPP^+^ (500 µM) for 24 h. Male C57BL/6J mice. MPTP (20 mg/kg) was subcutaneously injected daily for 5 days.	NaHS at 50, 100, 200, 400 µM administered 30 min prior MPP^+^. From days 4–8, NaHS was injected intraperitoneally (5.6 mg/kg) for 10 days, 30 min prior MPTP.	[40] [42]
	AD	3xTg-AD mice.	NaHS (0.5 mg/kg) was administered intraperitoneally once daily for 12 weeks starting at 6 or 12 months of age.	[47]
	Stroke	Sprague-Dawley rats. Adult male C57BL/6J mice subjected to tMCAO. 1) MCAO 2 h occlusion followed by reperfusion. 2) Hyperglycaemia MCAO model of tPA-enhanced haemorrhagic transformation.	NaHS (25 µmol/kg dissolved in 2.5 ml of saline) administrated via intravenous injection 1 and 30 min before reperfusion. NaHS (25 µmol/kg) was administered at the onset of reperfusion or when the tPA infusion began.	[52] [55]
	GBM	Rat C6 glioma cell line	NaHS (0.25, 0.5 and 1 mM) for 48 h	[58]
S-ASP (sulphur-aspirin hybrid) and S-DI (sulphur-diclofenac)	AD and PD	Primary human microglial cells, primary human astrocytes and cell lines: THP-1, U118, SHSY-5Y exposed to: 1. CBS inhibitor hydroxylamine 1 mM. 2. LPS at 1 µg/mL and IFN-γ at 333 U/mL.	S-ASP and S-DI administered at 1,3, 10, 30, and 50 µM for 2, 4, 8, or 12 h.	[101]
Sodium sulphide (Na_2_S)	AD	SHSY-5Y exposed to HEWL aggregates (lysozyme)	Na_2_S (12 mM) was administered in a molar ratio of 1:5 (HEWL:H_2_S) for 24 h.	[48]
	Stroke	1. Sprague-Dawley rats subject to MCAO. 2. C57BL/6 mice subject to acute cerebral anoxia.	1. Rats received Na_2_S (5, 10, 20, 40 mg/kg) via intravenous injection within 15 min of MCAO. 2. Mice received Na_2_S (7.5, 15, 30 mg/kg) via intravenous injection within 15 min after hypoxic anoxia.	[102]
	GBM	Human T98G cells Human U87 cells	Na2S (476 µM) for 4 h.	[57]
S-Propargyl-cysteine	AD and PD	Male Sprague-Dawley rats subjected to LPS bilateral intracerebroventricular injection.	S-Propargyl-cysteine (20, 40, 80 mg/kg) administered once per day three days before LPS insult and thereafter continuously for 9 days.	[50]
Sulphur water	AD	3xTg-AD mice.	Sulphurous water (thermal water with high H_2_S content, 125 mg/L) was administered intraperitoneally (12 mL/kg) daily for 12 weeks.	[47]
Tacrine-H_2_S	AD	Kunming mice subjected to aluminium chloride (AlCl_3_) model of AD by intraperitoneal injection (AlCl_3_: 100 mg/kg), every other day for 50 days.	Tacrine-H_2_S: (5 and 15 mmol/kg/day) via intraperitoneal injection for 20 days after AlCl_3_ treatment.	[103]

**Fig. 5:**
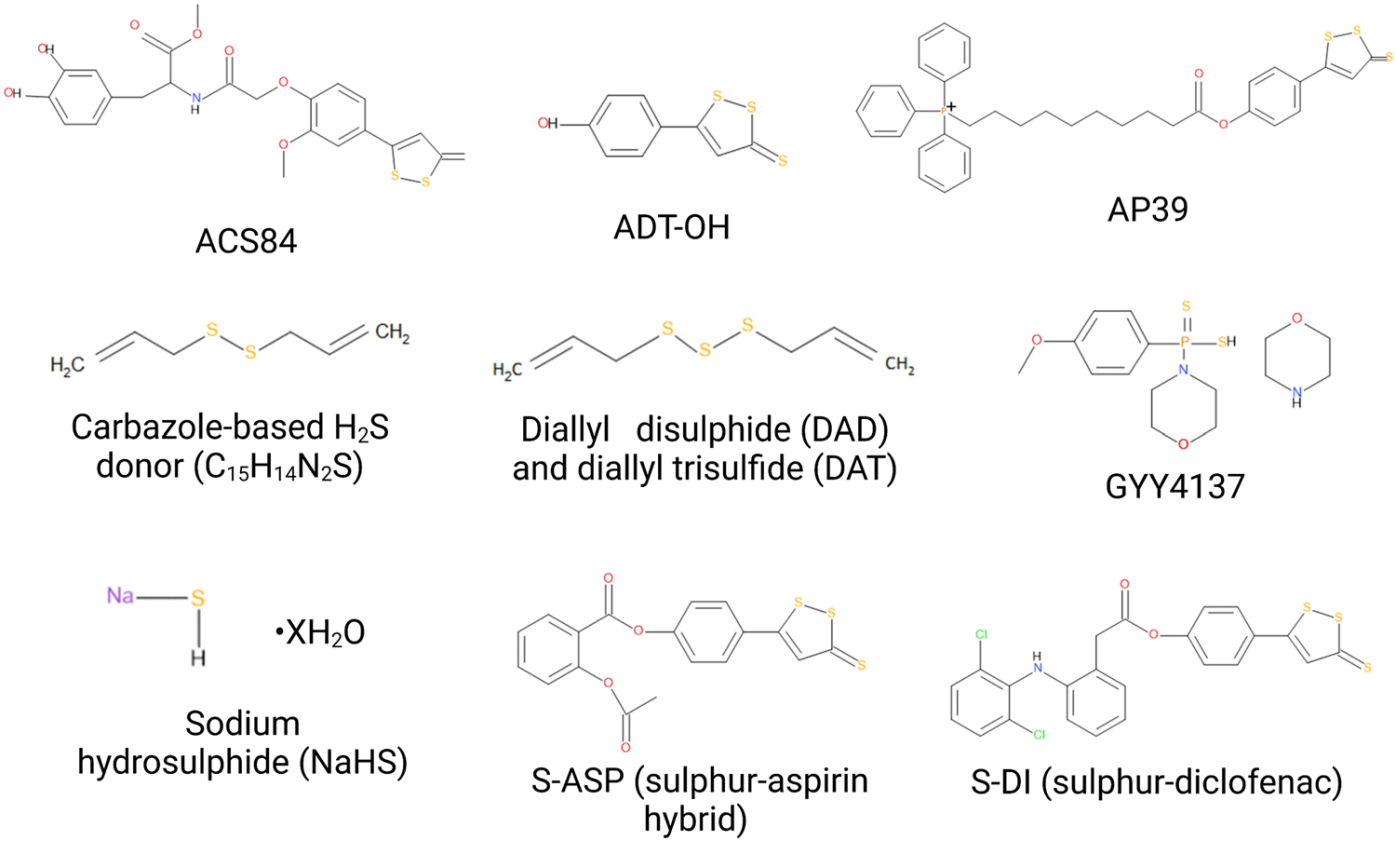
Biochemical structures of ACS84, ADT-OH, AP39, Carbazole-based H_2_S donor (C_15_H_14_N_2_S), Diallyl disulphide (DAD) and diallyl trisulfide (DAT), GYY4137, Sodium hydrosulphide (NaHS), S-ASP (sulphur-aspirin hybrid) and S-DI (sulphur-diclofenac)

Biochemical structures of ACS84, ADT-OH, AP39, Carbazole-based H_2_S donor (C_15_H_14_N_2_S), Diallyl disulphide (DAD) and diallyl trisulfide (DAT), GYY4137, Sodium hydrosulphide (NaHS), S-ASP (sulphur-aspirin hybrid) and S-DI (sulphur-diclofenac) are depicted in Fig. 5.

## Perspectives: Overcoming Limitations in H_2_S Delivery

Thus far, H_2_S donors have not been specifically targeted to the brain even when indicated in neurological dysfunction. Drugs with molecular weight below 500 Da and high lipophilic content usually diffuse the BBB easily [104] thus forth, H_2_S donors targeting the brain must possess such qualities. Carrier-mediated transport and receptor-mediated endocytosis/transcytosis have been explored and, more recently, biomaterial-mediated strategies have been envisaged, including polymeric-based systems, already approved by the Food and Drug Administration Agency. These polymeric systems include polylactide, polycaprolactum and polyglycolide that can be employed as hydrogels and nanoparticle formulations. Another approach has been the use of liposomes. These structures, due to their similarity with the lipid bilayer in cellular membranes along with CNS targeting potential offers an interesting approach when exploring CNS drug delivery. In particular, approaches embedding antibodies within liposome structure, also referred as immunoliposomes, have been explored in the treatment of AD [105].

Nanoparticle formulations based on polymer or liposomal structures may also assist drug movement across the BBB when CNS-related drugs cannot cross the BBB on their own. In other scenarios of human disease, preclinical studies of nanoparticle delivery of drugs have reported that nanoparticles could not only increase the compounds half-life time but also decrease the amount of drug being eliminated by the reticuloendothelial system, thus, improving stability and increasing drug exposure [106, 107]. Recently, in our laboratory, we have explored nanoparticle approaches to encapsulate H_2_S donors, protecting from rapid degradation and release of H_2_S whilst exploring different routes of administration aiming to reduce administration regimes [108–110]. Whilst observations must be confirmed in an in vivo setting, it was noted that liposomes employed to carry ADT-OH across murine skin observed a delayed flux across the skin compared to formulations without liposomes suggesting liposomes may be beneficial in giving a controlled release of drug [108]. Furthermore, cationic liposomes used in the controlled release of H_2_S from sodium thiosulphate were able to retain its biological properties in hypoxia-like environment [110]. Finally, PLGA nanoparticles carrying sodium thiosulphate observed a sustained cellular drug uptake whilst maintaining the pro-angiogenic potential of H_2_S [109]. These new approaches are supported by other researchers exploring the use of mesoporous iron oxide nanoparticle (MION) with polyethylene glycol (PEG) and lactoferrin as carriers for the H_2_S donor DATS [111]. Another relevant aspect of this novel method of H_2_S delivery is the fact that MION, due to their excellent superparamagnetism, enable non-invasive in vivo tracing through MRI techniques, that can be further applied as a means to measure pharmacokinetic parameters such as drug distribution [111].

Nonetheless, emerging pharmaceutical technology to successfully deliver H_2_S at consistent rates, limiting its degradation and successfully targeting the CNS is in its infancy. It is therefore paramount to encourage interdisciplinary collaborations bringing together the biochemistry and pharmacological aspect of H_2_S metabolism within the CNS field to offer better approaches and methods in the investigation of H_2_S-based therapeutics for neurological health.

## Conclusion

H_2_S is an important mediator in nervous system physiology playing a protective role in most neurological disorders. H_2_S elicits anti-oxidative, anti-apoptotic, and anti-inflammatory properties in the CNS which may ameliorate a range of neurological disorders. This review highlights the potential therapeutic application of H_2_S-based compounds in treating neurological disease. Although many H_2_S releasing drugs have been tested in scenarios resembling neurological impairments (in vitro and in vivo), many limitations in the translation to the clinic are present, including the gaseous nature of H_2_S compounds, the rapid release of H_2_S from these molecules and lack of targeting to the CNS, limiting the successful crossing of the BBB. Recent approaches including chemical modifications to the H_2_S donor and nanoparticle formulations (both polymer- and liposome-based) to encapsulate H_2_S-based compounds may offer a solution to protect these compounds from rapid degradation, control their rates of release whilst providing targeting opportunities to reach and cross the BBB. Furthermore, we believe exploring additional routes of drug delivery such as buccal or transdermal drug delivery would aid patient self care and reduce the need for medical intervention. Understanding these new avenues in pharmacological formulations and enhancing interdisciplinary research may result in the generation of a suitable H_2_S donor treatments to address neurological conditions.

## Data Availability

Enquiries about data availability should be directed to the authors.
